# Retinal Gene Therapy: Surgical Vector Delivery in the Translation to Clinical Trials

**DOI:** 10.3389/fnins.2017.00174

**Published:** 2017-04-03

**Authors:** G. Alex Ochakovski, K. Ulrich Bartz-Schmidt, M. Dominik Fischer

**Affiliations:** ^1^Centre for Ophthalmology, University Eye Hospital, University Hospital TuebingenTuebingen, Germany; ^2^Nuffield Laboratory of Ophthalmology, Department of Clinical Neurosciences, University of OxfordOxford, UK

**Keywords:** gene therapy, vitreo-retinal surgery, retina, AAV, blindness, monogenic disorders

## Abstract

An exceptionally high number of monogenic disorders lead to incurable blindness, making them targets for the development of gene-therapy. In order to successfully apply therapeutic vector systems *in vivo*, the heterogeneity of the disease phenotype needs to be considered. This necessitates tailored approaches such as subretinal or intravitreal injections with the aim to maximize transduction of target cell populations, while minimizing off-target effects and surgical complications. Strategic decisions on parameters of the application are crucial to obtain the best treatment outcomes and patient safety. While most of the current retinal gene therapy trials utilize a subretinal approach, a deeper understanding of the numerous factors and considerations in choosing one delivery approach over the other for various ocular pathologies could lead to an improved safety and treatment efficacy. In this review we survey different vector injection techniques and parameters applied in recent retinal (pre-)clinical trials. We explore the advantages and shortcomings of each delivery strategy in the setting of different underlying ocular pathologies and other relevant factors. We highlight the potential benefits for patient safety and efficacy in applying those considerations in the decision making process.

## Introduction

A unique set of highly relevant features, conveniently combined in a single organ, have placed the eye at the forefront of gene therapy development. As a small, compartmentalized, and paired organ with excellent access both for intervention and high resolution functional and structural diagnostics, the eye benefits from the vast knowledge on the genetic basis of ocular disease in general and retinal dystrophies specifically. As multiple monogenetic causes have been identified to lead to blinding retinal disorders, there is a rich pool of potential targets for drug development.

From a surgeon's perspective, the eye is easily accessible using minimally invasive techniques with its transparent media allowing a direct view of the operating field. Standard techniques and instruments can be used to gain direct access to the inner contents of the eye. The vitreous and retina are well defined compartments within the eye. Considered as part of the brain, the retina consists of distinct layers in which ganglion cells and nerve fibers are situated closest to the pupil. These are followed by more distal interneurons. The light sensing photoreceptors (PRs) are found buried deepest in the neuroretina and interact directly with a monolayer of retinal pigment epithelium (RPE) cells, which in turn sit on top of the Bruch's membrane and the choroid with its rich capillary network. The surgeon has mainly two options to deliver vector solution to the retina, either by injecting it into the vitreous body which fills the core of the eye, using the so-called intravitreal (IVT) approach, or by injecting the solution under the sensory retina, in a potential space between the PRs and RPE, with a subretinal (SR) injection.

In many ocular genetic disorders in clinical trial phase, a consensus regarding the preferred injection type has already been reached. For example, in Leber's congenital amaurosis (*RPE65* gene) in which deeply situated RPE cells are the target of gene therapy, the SR approach is employed in all current trials (Table 1 in Supplementary Material). On the other hand, in Leber's hereditary optic neuropathy (LHON), a disease in which the more superficial layer of ganglion cells is targeted, the IVT approach has become the choice in all clinical trials. However, in several other cases, such as in macular dystrophies and in retinitis pigmentosa, both approaches have been suggested and no single consensus has emerged. Some genetic diseases, like X-linked retinoschisis and Stargardt disease, have only recently started to gain momentum toward translation into clinical trials and the most optimal injection method for these clinical entities may yet to be determined. In this mini-review we seek to provide a detailed assessment of the relevant factors and their impact on the decision matrix in order to facilitate and guide the decision-making process on future surgical protocols.

## Intravitreal injection

Intravitreal (IVT) injection is a widely-used technique to deliver therapeutic agents, the most common being drugs inhibiting vascular endothelial growth factors, antibiotics and glucocorticoids. IVT injections are one of the most commonly performed ocular surgery procedures in the developed world, second only to cataract surgery.

**Figure 1:**
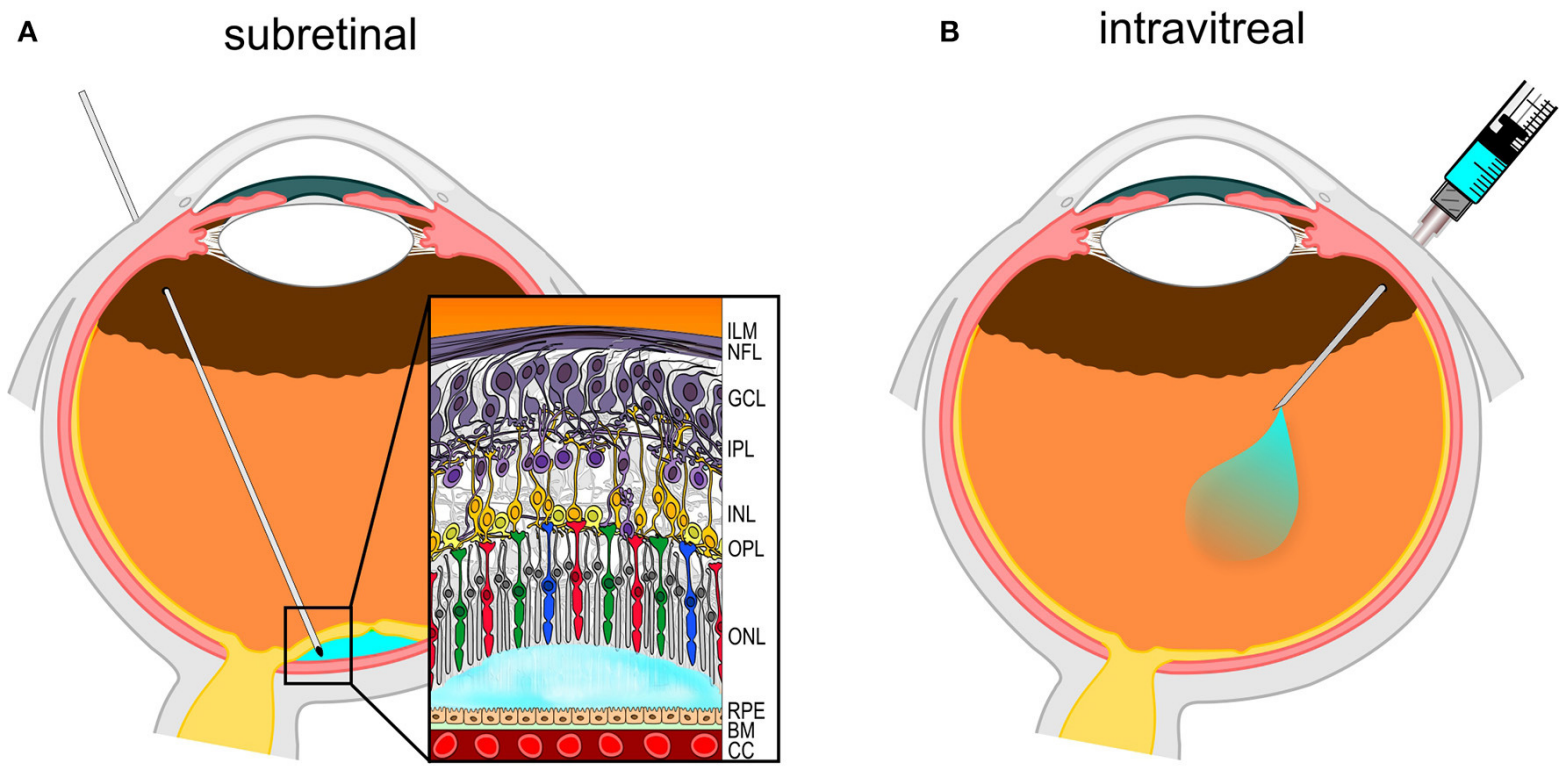
**Diagram of routes of surgical intraocular gene therapy delivery. (A)** Subretinal (SR) injection performed via the *pars plana*. The needle delivers the vector solution (in light-blue) into the potential space between retinal pigment epithelium (RPE) and photoreceptors in the outer nuclear layer (ONL). **(B)** Intravitreal (IVT) injection also uses the *pars plana* access to deliver vector solution into the vitreous cavity.

The procedure is generally performed under local anesthesia with e.g., lidocaine 2%. During the procedure, the eyelids and eyelashes are treated with disinfectant such as povidone-iodine solution. Subsequently, a 30 Gauge needle is inserted through the sclera at the *pars plana* region, 3.5–4 mm posterior to the limbus between vertical and horizontal muscles (Figure 1). The therapeutic agent is directly injected into the vitreous cavity with limited reflux (Boon et al., 2008; Xing et al., 2014).

Although considered relatively safe, IVT injections bear some degree of risk for complications. One of the major post-injection complications is endophthalmitis, with per-injection complication rates ranging between 0.021% (Dossarps et al., 2015) and 0.16% (Wu et al., 2008). The majority of patients with a history of endophthalmitis maintain reduced visual acuity in follow-up examinations (Dossarps et al., 2015). Other observed complications include: retinal detachment, iritis/uveitis and transient intraocular pressure elevation (Jager et al., 2004). The relative safety of IVT seemingly made many practices adopt less rigorous surgical hygiene standards. For example, 48% of the 765 surveyed retinal specialist in US reported wearing no gloves during an IVT injection (Green-Simms et al., 2011). Streptococcal isolates were found to be 3 times more common after IVT than after intraocular surgery (McCannel, 2011).

When considering the IVT injection in pre-clinical settings, key differences between the eyes of human and small animal models need to be accounted for, namely the vitreous volume and the lens/eye ratio. For example, the spherical lens of the rat occupies most of its eye, leaving only a volume of 13 μl that is occupied by the vitreous and thus restricting the effective IVT injection volume to 3–5 μl (Dureau et al., 2001). The lens takes up even more of the globe in mice, where IVT injections are generally limited to volumes of up to 2 μl (Lin et al., 2014; Rösch et al., 2014; Du et al., 2015). Rabbits on the other hand have larger eyes, yet their lens still occupies ~40% of the axial length of the eye (Trivedi et al., 2002), which allows IVT injections of up to 50–100 μl (Chen et al., 2011; Gasparin et al., 2014). From the surgical standpoint, big lenses considerably restrict the space in which the needle can be safely maneuvered without damaging the retina or the lens and its capsule. To minimize the risk for lens touch and cataract formation and/or retinal perforation, adjusted protocols have been proposed for trans-scleral or trans-retinal approaches (Chiu et al., 2007). In the context of gene therapy, pre-clinical experiments on non-human primates were designed to closely mimic the clinical administration of recombinant AAV (rAAV). Their findings suggest that extraocular biodistribution and shedding of rAAV vehicle after IVT injection is considerable, especially in blood and lymphatic tissue (Seitz et al., 2016). Consequently, a consistent humoral immune response against rAAV can be observed c. 7 days after IVT Injection (Reichel et al., 2016). Apart from these safety aspects, transduction efficiency of target cells is a key variable in the context of efficacy. When applying rAAV2 and rAAV8 (the most commonly serotypes in clinical use) intravitreally, both serotypes show limited transduction efficiency confined to inner retinal cells (Li et al., 2008; Igarashi et al., 2013). One study showed two orders of magnitude lower transduction of whole retina after IVT of rAAV8 compared to subretinal injection in non-human primates (Seitz et al., 2016).

## Subretinal injection

Unlike IVT, subretinal (SR) injections constitute “proper” ophthalmic surgery performed by vitreo-retinal surgeons. SR maneuvers are routinely used in severe cases of submacular hemorrhage or other complex vitreoretinal disease involving the subretinal space. In clinical research, subretinal surgery has been performed in macular translocation surgeries (Aisenbrey et al., 2002), electronic (Zrenner et al., 2011), or stem-cell implants (Schwartz et al., 2015) and gene therapy trials (Bainbridge et al., 2008; Hauswirth et al., 2008; MacLaren et al., 2014; Banin et al., 2015; Fischer et al., 2016a), with the aim to prevent or reverse blindness.

The SR injection can be performed under retro-/parabulbar anesthesia (Hauswirth et al., 2008) or under general anesthesia in an operating theater. After disinfection, a three-port *pars plana* vitrectomy is performed, mostly using standard 23 or 25G trocar systems. After successful detachment of the posterior hyaloid membrane and removal of the vitreous, e.g., a double-barrelled 23G needle with 41G tip is inserted through the trocar. The tip is guided to the subretinal area and a small infusion of balanced salt solution (BSS) is performed into the potential subretinal space to form a bleb. Once the subretinal space has formed and location of the bleb is within the targeted region (Figure 1), the same retinotomy (injection channel through neuroretina) is used to guide a second instrument with the same tip built into the subretinal space for the injection of the therapeutic agent using a controlled flow rate (Fischer et al., 2016b).

The described approach is the “two-step” variant of the SR injection technique first described by Bainbridge et al. (2008) and employed by MacLaren et al. (2014) and Fischer et al. (2016b). Using this approach, a sub-retinal bleb of BSS solution is induced before actual vector injection takes place. This opens the potential subretinal space and the surgeon can ensure the correct plane has been chosen and that the bleb is located and traveling toward the targeted location. In contrast, the “single-step” injection used by Maguire et al. (2008), aims to place the vector solution directly in the subretinal space while the retina is still fully attached. The “two-step” approach offers several advantages like the possibility to better assess the direction of bleb spread as well as minimize vector loss by misguided injection (e.g., accidental delivery into vitreous, subhyloidal or suprachoroidal space).

Vitrectomy as part of the SR injection procedure is associated with general surgical complications, including increased rate of cataract development (Bennett et al., 2016) while the subretinal injection *per se* may induce a limited degree of outer nuclear layer (ONL) thinning (Jacobson et al., 2006). A few mild and self-resolving subconjunctival and retinal hemorrhages were also observed (Rakoczy et al., 2015) as well as a case of acute endophthalmitis which resolved under antibiotic treatment (Schwartz et al., 2015). An intraoperative macular hole has been reported as well (Campochiaro et al., 2017). In contrast to IVT injections, the amount of currently available data on complications in SR injections is too limited in order to reliably estimate the complication rates. Nonetheless, much development effort has been concentrated to provide technical solutions that will enable safer SR injections, such as the use of intra-operative optical coherence tomography (OCT) imaging (Ehlers et al., 2014) for live head-up-display visualization of the injection bleb as well as surgical robotic solutions (Meenink et al., 2013) that facilitate fine movements in the eye and eliminate hand-tremor effects.

In pre-clinical settings, *ab externo* SR injection has been successfully tested in mice (Fischer et al., 2013), rabbits (Peden et al., 2011; Martorana et al., 2012), and pigs (Smet et al., 2012). The procedure involves penetration of the conjunctiva, sclera, choroid, and RPE at the equatorial region in order to reach the subretinal space without disrupting the retina. A fine needle is advanced at an angle to enter the subretinal space, while not penetrating the retina with the tip of the needle. With the bevel facing the photoreceptor outer segments, volume can be injected to detach a large portion of retina in one injection. *Ab externo* approaches can cause escape of any amount of the injected volume into the suprachoroidal space, choroid or even orbita. Because more sturdy instruments are necessary to penetrate the sclera, the injection channel is usually larger than in a trans-retinal approach, where extremely fine tips made by flexible polytetrafluoroethylene are used. The larger injection channels translate into potentially larger volume of reflux. Sparing the retina was possible in most cases using such an approach in pigs. Yet 13% of the 124 performed procedures in one study resulted in iatrogenic retinal perforations (Smet et al., 2012).

## Gene delivery vector

The most common vector currently being used in retinal gene therapy trials is a modified adeno-associated virus (AAV), which offers numerous advantages, such as a high transduction efficiency in both, dividing and non-dividing retinal cells, a range of natural serotypes with complimentary tropism favoring various cell populations (Ellis et al., 2013) and a good safety profile (Trapani et al., 2014). In this text we mainly focus on AAV as the main vector in our examples and reasoning.

## Anatomical layout of target tissue

In each setting, multiple factors determine the best surgical approach for vector delivery. To facilitate implementation in decision matrix, individual factors will be highlighted independently. The layout of the target tissue to be treated is maybe the most obvious determinant of a preferred delivery route. Considering the vertical lamination of the retina and its neighboring tissues, IVT approaches would be predicted more successful in transducing ganglion and/or bipolar cells populating the inner retina. While diseases primarily affecting RPE and/or photoreceptors may be most efficiently targeted by SR injections. Recently, considerable research efforts have been invested into developing ways to minimize the impact of this factor on the decision outcome, such as development of mutant AAV vector capsids capable of reaching the photoreceptors also by IVT administration (Dalkara et al., 2013; Kay et al., 2013). New developments in vector design could potentially provide additional flexibility (Dalkara et al., 2013; Cronin et al., 2014).

A second anatomical consideration is the non-homogenous cell density distribution across the surface of the retina and its implications. The fact that cones have a sharp density peak in the central fovea, as opposed to rods, which reach highest density at 20°, has important functional and surgical implications (Purves et al., 2001). The cone-mediated central visual function would be the most relevant target for localized gene therapy if RPE or cone photoreceptors are targeted or to prevent secondary cone degeneration, because a therapeutic effect in the central 10° visual field would have the most substantial impact on the quality of life of the patient compared to a treated peripheral island of comparable size. When targeting a subpopulation (rods vs. cones), the spatial density gradients become especially relevant, as in the case of *CNGA3* gene therapy for achromatopsia, where cone photoreceptors are targeted at the area of their highest physiological density with potentially the most significant impact on patients' quality of life (Banin et al., 2015).

Thirdly, the tempero-spatial pattern of disease progression is also highly relevant. For example, in rod-cone dystrophies progression is centripetal, with central vision being the last to be affected by the disease. Whereas, in Stargardt's disease, central vision is affected early on, as disease progresses in a more centrifugal fashion. Since gene therapy can only be successful if the target cells are still viable, disease type, and stage need to be considered when choosing the application approach.

These considerations usually have to be assessed as an integrated matrix of factors in clinical scenarios. For example, choroideremia, a progressive degenerative disease is caused by genetic mutation or deletion of *CHM* on the X-chromosome (van den Hurk et al., 1997). This leads to a degeneration of the choroid, retina and RPE, to the extent where the intact area that can potentially be treated in advanced stages is very limited (Barnard et al., 2015). In such cases a SR injection is very suitable approach to efficiently target the remaining cell groups of the outer retina and RPE, while limiting the delivery of the vector to the treated area and avoiding off-target exposure and minimizing systemic spread. On the other hand, in earlier stages of choroideremia, most of the retina is still intact and could potentially benefit from gene-therapy. An IVT approach in this case could in principle be advantageous, as broader areas of retinal tissue could be treated. All six listed CHM gene therapy trials have employed the SR approach targeting patients with advanced disease (Table 1 in Supplementary Material).

Conversely, mutations in mitochondrial DNA at position 11,778 cause ganglion cells to degenerate, leading to LHON (Man et al., 2002). In order to reach a great number of ganglion cells at the surface of the inner retina, an IVT injection might be a better option. This reasoning has likely driven the selection injection method for LHON in current clinical trials, as all five listed trials used this approach (Table 1 in Supplementary Material).

## Anatomical barriers and their integrity

The internal limiting membrane (ILM) likely acts as a barrier for the diffusion of AAV vectors between the vitreous and the retina (Dalkara et al., 2009). This property of the ILM can become a major obstacle to treatment delivery or alternatively be harnessed as a key component to achieve the therapeutic goals by choosing the right injection method.

In IVT injection, ILM is thought to prevent the diffusion of AAVs and transduction of target cells in the outer retina and has been a major obstacle in the development of retinal gene therapy for retinal dystrophies primarily affecting the RPE and/or photoreceptors. Several methods that target ILM integrity, like laser-based photocoagulation (Lee et al., 2013), surgical peeling (Takahashi et al., 2017), and enzymatic lysis (Dalkara et al., 2009) of ILM have been demonstrated to facilitate vector transduction of the target retinal tissue via the ILM, yet not without potential risk for complications. In the case of SR injection, the ILM likely acts as a natural barrier that helps prevent unwanted vector spread to the vitreous, anterior segment and systemic circulation.

To benefit from the compartmentalization of the retina rather than try to overcome it, the surgical approach should be optimized for the anatomical position of the target cell. With the current vector systems, the SR delivery seems better suited for targeting RPE or photoreceptor layers, whereas IVT injection is optimal for targeting inner retinal neurons or Müller glia.

## Multiplicity of infection

The ratio between the total number of vector particles to the number of cells potentially transduced, also known as multiplicity of infection (MOI) is known to be correlated with the treatment effect in gene therapy (MacLaren et al., 2014). Using the IVT delivery approach, an increased amount of off-target cells will be exposed to the vector in comparison with an SR injection of the same total dose. This greater number of cells would reduce the MOI in an IVT approach when comparing it to a SR injection at a fixed dose. If this would correlate with efficacy measures in a dose response function, IVT approach needs to apply significantly higher numbers of vector particles to achieve the same MOI of an SR approach. This can be achieved by using a higher viral concentration and/or higher injection volume. Yet, increasing the total vector dose would increase the risk for unwanted shedding and more pronounced biodistribution, which in turn increases the likelihood of a potentially harmful immune response.

## Immune response and vector re-administration

Injections to different ocular compartments have been shown to results in different humoral immune responses in mice (Li et al., 2008) and monkeys (Seitz et al., 2016). IVT injections triggered a humoral immune response to the AAV vehicle while SR injections didn't cause a humoral response nor did they affect a repeated administration in the partner eye (Li et al., 2008, 2009). Further studies in non-human primates (NHP) have shown a dose dependent immunologic response also in SR injection (Ye et al., 2016). Pre-existing anti-AAV antibodies in NHPs strongly correlated with weak trans-gene expression when AAV was delivered using the IVT approach (Kotterman et al., 2015). In contrast, SR injected AAV effectively transduce retinal cells despite the presence of neutralizing anti-AAV antibodies in the serum and intraocular fluid (Amado et al., 2010).

## Systemic vector shedding

The eye is considered an “immune privileged” organ, a property that has allowed such immunologically challenging procedures as transplants to be performed without systemic immunosuppression in case of the cornea (Niederkorn, 2013). The rest of the body (except the brain) does not have such a status and therefore has a higher potential for inflammatory response to the injected vector. Avoiding systemic spread of AAV vector from the eye to the rest of the body is also important to minimize the formation of neutralizing antibodies, which have been proven to be very efficient against AAV even at low titers, leading to complete neutralization of vector transduction in some cases (Manno et al., 2006). AAV vector has been detected in larger quantities and for longer time periods in all bio-fluids following an IVT injection in comparison with SR administration (Seitz et al., 2016).

## Biomechanic stability of the retina

The effect of the underlying disease on the structural health of retina has to be considered, especially when it comes to SR injections. Fragile retinas might fail to maintain their structural integrity when exposed to mechanical tension induced by the injection bleb. To minimize the risk of iatrogenic macular hole formation, injection velocity and pressure should be tightly regulated to avoid water-jet effect in the small diameter needle and allow gradual bleb formation.

## Retinal adhesiveness

Several underlying pathological conditions can alter the adhesive force between outer retina and the RPE. Both, too strong and too weak retinal adhesion could lead to complications during a SR injection. Patients with generally elevated risk for retinal detachment (1993; Polkinghorne and Craig, 2004) like cases of previous detachment, extreme myopia, family history of detachment could benefit from a tailored risk and suitability assessment for a SR injection. Moreover, the genetic condition in question *per se* should be evaluated for its effect on the adhesiveness of the retina in the treated area (Le Meur et al., 2006). As an example, one of the more recent targets in gene therapy trials, the X-linked juvenile retinoschisis, predisposes for retinal detachment, and vitreous hemorrhage and thus might not be the optimal disease for SR injection (George et al., 1995; Lee et al., 2009). On the other hand, more peripheral degenerations like those seen in classical retinitis pigmentosa (RP) cases are very unlikely to cause retinal detachment. Strong adhesive force between retina and RPE might lead to difficulties inducing the bleb in SR injection, prolonging the time of intervention and increasing the risk for complications.

## Conclusion

The SR and IVT injection are two valid surgical delivery approaches in gene therapy, yet substantial differences between the two methods in numerous aspects should be taken into consideration. SR injection is generally the preferable approach when the outer retinal layers are targeted, especially when treatment area is limited and localized, to harness the immunologic benefits of vector spread restriction and in cases where re-administration in same or partner eye is a likely option. One obstacle of the SR approach is the learning curve and high manual dexterity required from the operating retinal surgeon and the potential for damage during the transretinal manipulation, yet advancements in ocular surgery robotics development might help overcome this hurdle in the near future (NCT03052881). IVT injection is advantageous when inner retinal layers and wide areas of the retina are to be treated, in particular when retinal structural resilience is compromised through the underlying or concomitant diseases and where no major concerns over systemic shedding and the off-target transduction effect are present. As mentioned previously, the low transduction efficiency and considerable shedding of rAAV serotypes 2 and 8 together with the humoral immune response that follows thereafter, have been major hurdles that keep the IVT from becoming the preferred injection method for ocular gene therapy. Ongoing improvements in vector design could help overcome those limitations and potentially make IVT both safe and efficient option for gene therapy over the long run (Dalkara et al., 2013; Kay et al., 2013).

## Author contributions

GO and MF reviewed literature, GO and MF wrote manuscript, KB reviewed manuscript.

## Funding

MF is consultant to NightstaRx (Wellcome Trust Building, 215 Euston Road, London, UK), a gene therapy company established by the University of Oxford and funded by the Wellcome Trust. MF has received funding from the Tistou and Charlotte Kerstan Stiftung, ProRetina, the UK Medical Research Council (HMRXDS0), and the Henning-Zügel Stiftung.

### Conflict of interest statement

The authors declare that the research was conducted in the absence of any commercial or financial relationships that could be construed as a potential conflict of interest.
